# Epibiotic Bacteria Isolated from the Non-Indigenous Species *Codium fragile* ssp. *fragile*: Identification, Characterization, and Biotechnological Potential

**DOI:** 10.3390/microorganisms12091803

**Published:** 2024-08-30

**Authors:** Wafa Cherif, Leila Ktari, Bilel Hassen, Amel Ismail, Monia El Bour

**Affiliations:** National Institute of Marine Sciences and Technologies (INSTM), University of Carthage, Tunis 2025, Tunisia; cherifwafa@yahoo.fr (W.C.); hassenbilel70@gmail.com (B.H.); ismail_ml@yahoo.fr (A.I.)

**Keywords:** epibiotic bacteria, alga, *Codium fragile*, invasive, 16S rRNA, agarolitic, antibiotic resistance

## Abstract

Due to their richness in organic substances and nutrients, seaweed (macroalgae) harbor a large number of epiphytic bacteria on their surfaces. These bacteria interact with their host in multiple complex ways, in particular, by producing chemical compounds. The released metabolites may have biological properties beneficial for applications in both industry and medicine. In this study, we assess the diversity of culturable bacterial community of the invasive alga *Codium fragile* ssp. *fragile* with the aim to identify key groups within this epiphytic community. Seaweed samples were collected from the Northern Tunisian coast. A total of fifty bacteria were isolated in pure culture. These bacterial strains were identified by amplification of the ribosomal intergenic transcribed spacer between the 16S and the 23S rRNA genes (ITS-PCR) and by 16S rRNA sequencing. Antimicrobial activity, biochemical, and antibiotic resistance profile characterization were determined for the isolates. Isolated strains were tested for their antimicrobial potential against human and fish bacterial pathogens and the yeast *Candida albicans*, using the in vitro drop method. About 37% of isolated strains possess antibacterial activity with a variable antimicrobial spectrum. Ba1 (closely related to *Pseudoalteromonas spiralis*), Ba12 (closely related to *Enterococcus faecium*), and Bw4 (closely related to *Pseudoalteromonas* sp.) exhibited strong antimicrobial activity against *E. coli*. The isolated strain Ba4, closely related to *Serratia marcescens*, demonstrated the most potent activity against pathogens. The susceptibility of these strains to 12 commonly used antibiotics was investigated. Majority of the isolates were resistant to oxacillin, cefoxitin, tobramycin, and nitrofurantoin. Ba7 and Ba10, closely related to the *Vibrio anguillarum* strains, had the highest multidrug resistance profiles. The enzymes most commonly produced by the isolated strains were amylase, lecithinase, and agarase. Moreover, nine isolates produced disintegration zones around their colonies on agar plates with agarolitic index, ranging from 0.60 to 2.38. This investigation highlighted that *Codium fragile* ssp. *fragile* possesses an important diversity of epiphytic bacteria on its surface that could be cultivated in high biomass and may be considered for biotechnological application and as sources of antimicrobial drugs.

## 1. Introduction

Algal surfaces provide suitable substrates for microbial colonization and secrete various organic substances that serve as nutrients for bacterial growth and microbial biofilm formation. Microbial communities living on the seaweed surface are highly complex and consist of a dynamic group of microorganisms, including bacteria, fungi, diatoms, protozoa, spores, and the larvae of marine invertebrates. These organisms play crucial roles in every marine ecological process, hence the growing interest in studying their populations and functions [1,2]. Novel and diverse molecules with a large antimicrobial spectrum derived from algae and their associated bacteria could have a huge potential for use in biotechnological research. Different studies have reported that the micro- and macro-organisms of an opportunistic nature colonize surfaces of seaweed, producing protective compounds [3]. Surface-associated marine organisms such as microbes, diatoms and larval forms of marine invertebrates have been detailed to be related with algal thallus [4,5]. These epibiotic communities, especially epiphytic bacteria, provide hormones, vitamins, minerals, carbon dioxide, and a large number of bioactive metabolites to the seaweed and therefore play an important role in the morphogenesis, growth, and immune defense. In return, the seaweed provides a habitat, oxygen, and carbohydrates such as algal polysaccharides to the associated microorganisms [6,7]. The importance of microbial diversity on the macroalgae surface, especially among the bacterial genus, is highly host-specific as new species emerge from this algal environment [8]. The secondary metabolites produced by these bacteria are widely recognized for their importance in biotechnological uses [9,10,11]. Antimicrobial properties [12] and antifungal activities [13] of the epiphytic bacterial communities from the macroalgae collected from the Tunisian coasts have been reported.

The green alga *Codium fragile* ssp. *fragile* (CFF) has spread rapidly in temperate areas throughout the globe from its native range in Japan [14]. This alien algae were observed for the first time on the Tunisian coasts in 1985 [15]. The distribution of the algae on the Tunisian coasts has since expanded over the last decade [16,17]. Studies on the bacteria associated with algae from the Tunisian coasts have gradually emerged over the last ten years; but none has targeted the invasive algae *Codium fragile*. As an invasive algae, having made various adaptations to settle into their new environment, *Codium fragile* have absolutely hosted a specific epiphytic bacterial community to protect themselves from exogenous pathogens [18]. This specific bacterial community could also be a source of new bioactive compounds of biotechnological interest. The current study has been undertaken based on the occurrence of the invasive seaweed *Codium fragile* on the northern coast of Tunisia to evaluate its associated bacteria and potential biotechnological application. After the isolation of culturable bacterial strains from the algal surface, a molecular identification of *Codium fragile*-associated bacteria was undertaken, and antimicrobial activity against various pathogens was evaluated. In addition, antibiotic resistance and enzymatic production profiles were performed. The main objectives of this study were to isolate culturable *Codium fragile*-associated bacteria and evaluate their biological activities, including enzyme production capacity, antibacterial activity, and antibiotic resistance, in order to provide additional data on invasive seaweeds collected from the Southern Mediterranean coasts.

## 2. Materials and Methods

### 2.1. Sampling Site

CFF samples were collected in January 2022 from a rocky shore at the La Marsa site (36°53′06″ N and 10°20′14″ E) in the northern coast of Tunisia (Southern Mediterranean Sea). The whole thalli were sampled carefully with their holdfasts by snorkeling at a 2 m depth. The freshly collected samples were immediately transported in an ice box to the laboratory and kept at 4 °C until analysis.

### 2.2. Taxonomic Identification of Seaweed Sample

**Figure 1:**
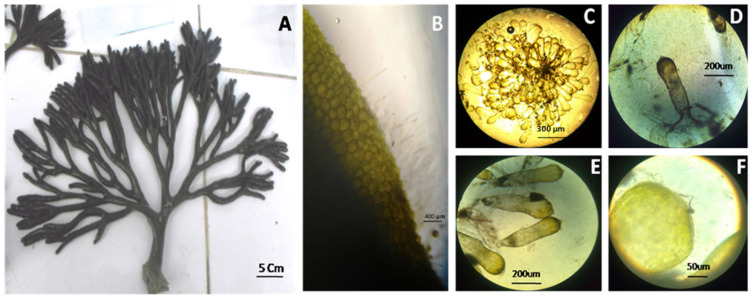
Photographs of *Codium fragile* subsp. *fragile*. (**A**) Dichotomous thallus with a spongy base, (**B**) surface covered with a multitude of hairs (**C**), branched cords composed of multiple utricles ending in a mucron, (**D**,**E**) utricles in the cross-section of the thallus, (**F**) Close-up view of long and pointed mucron on top of the utricle.

In the laboratory, the algal material was cleaned with seawater to remove the maximum number of epiphytes, and then kept in 2% formaldehyde seawater. Fixed materials were identified using a binocular microscope (Alphaphot-2.YS2-H; Nikon, Tokyo, Japan). The identification was based on different taxonomical keys. To determinate the subspecies of *Codium fragile*, the morpho-anatomical details were analyzed following the keys reported in the literature [19,20,21]. The green invasive alga *C. fragile* ssp. *fragile* (CFF) has a spongy, irregularly dichotomous branched thallus. The internal structure is composed of intertwined colorless medullary filaments that are amorphously cylindrical, and a green palisade-like layer of vesicles called utricles prolonging into a long pointed mucron. The presence of the pointed mucron and surface covered with a multitude of hairs, which gives it this aspect fluffy characteristic of *C. fragile*, confirmed the identity of the species (Figure 1).

### 2.3. Isolation of Seaweed Associated Bacteria

Fresh seaweed transported to the laboratory in sterile plastic bags were first rinsed with sterile seawater for a few seconds to remove the non-attached bacteria. Ten grams of seaweed were weighed and homogenized with 9 mL of sterile seawater, vortexed, serially diluted and 0.1 mL aliquots were plated on agar in triplicate, according to Ismail et al. [12]. The plates were incubated at 20 °C for a minimum of 7 days on Zobell Marine Agar (HiMedia) [22]. The individual bacterial strains were then isolated by repeated streaking. The pure cultures were stored at −80 °C in Marine Broth supplemented with 20% glycerol until further study.

### 2.4. Morphological Characterization

Morphological characterization tests were conducted based on the colony morphology, Gram staining, and catalase and oxidase tests, according to Dalton et al. [23].

### 2.5. Genotypic Characterization and Identification of Isolated Strains

The bacterial DNA was extracted using the Easy-Pure Bacteria Genomic DNA Kit (Trans-Gen Biotech, Beijing, China). Bacterial strain identification at the species and inter-species levels were performed via PCR 16S-23S intergenic spacer region amplification and using primers ITS-F (3′-GTCGTAACAAGGTAGCCGTA-5′) and ITS-R (3′-CTACGGCTACCTTGTTACGA-5′) as previously described by [24].

All the amplification products were visualized on a 1% agarose gel stained with 5 µL of SYBR Green (Atlas Clear-Sight DNA Stain, Bio-Atlas, Istanbul, Türkiye). ITS profiles were visually analyzed to group together the bacterial isolates exhibiting the same band pattern. At least one representative for each ITS group was identified by partial 16S rRNA sequencing. The amplification of the bacterial 16S rRNA gene was performed using the universal primers F27 (5′-AGAGTTTGATCCTGGCTGGCTCAG-3′) and R1492 (5′-TACGGCTACCTTGTTACGACTT-3′). Subsequent alignment of the sequence was performed on the NCBI database (http://www.ncbi.nlm.nih.gov/BLAST/Blast.cgi (accessed on 22 June 2023)).

A phylogenetic dendrogram was constructed using the neighbor-joining method, and tree topology was evaluated by bootstrap analysis of 1000 data sets using MEGA 6 [25].

The partial 16S rRNA gene sequences of each isolate were submitted to the NCBI GenBank database under the following accession numbers: ON908586, ON908587, ON908588, ON908589, ON908590, OR139885, ON908592, ON908593, ON908594, ON908595, ON908596, ON908597, ON908598, ON908599, ON908604, ON908609, ON908608, ON908605, ON908600, ON908601, ON908603, ON908602, ON908607, ON908606, OR139886, OR139887, OR139888, OR139889, OR139890, OR139891.

### 2.6. Antibiotic Resistance Profile Analysis

Antimicrobial susceptibility testing was performed on 24 isolates by the disk diffusion method on Mueller Hinton (MH, BIO RAD, Hercules, CA, USA) plates [12]. Twelve antimicrobial agents (BIO RAD, Marnes-la-Coquette, France) were tested (Table 1). A strain was determined to be sensitive to an antimicrobial compound if any growth inhibition zone was observed around the disk. The interpretation was performed in accordance with the French Society of Microbiology’s norms [26].

#### Determination of MAR Index

Determination of the multiple antibiotic resistances (MAR) index was calculated according to [27], using the following formula:

MAR = a/b

in which (a) is the number of antibiotics to which an isolate is resistant and (b) is the total number of the antibiotics used in the study.

### 2.7. Screening of Qualitative Enzymatic Production of CFF-Associated Bacteria

Several biochemical and enzymatic tests like the lipase, DNase, lecithinase, amylase, hemolysis, gelatinase, chitinase, cellulase, and agarose tests were carried out. All tests were performed in triplicate.

#### 2.7.1. Lipase

Bacteria were streaked on agar plates beforehand prepared with Tween-80. The results of this test were readable after 24 h at 37 °C, through the formation of an opaque zone denoting a lipase positive response [28].

#### 2.7.2. DNase

After incubation for 48 h at 28 °C, the surface of the agar medium (with a single colony) was covered with the toluidine blue reagent. A pink halo around the culture was formed in a DNase positive response [29].

#### 2.7.3. Lecithinase

Bacterial streaks were inoculated on nutrient agar containing a sterile egg yolk emulsion, followed by an incubation of 24 to 72 h at 30 °C. Opaque areas in the transparent halo would indicate degradation of the egg yolk lecithin by bacterial production of the lecithinase enzyme [30].

#### 2.7.4. Amylase

Bacteria were inoculated on specific starch agarin a single streak, and then incubated for 24 to 72 h at 30 °C. The hydrolysis of amylases was indicated by the presence of a clear zone around the colonies after the addition of Lugol. The absence of staining around the culture would show the degradation of starch (amylase-positive bacteria), while areas containing starch would stain brown (amylase-negative bacteria) [31].

#### 2.7.5. Hemolysis

Agar plates were prepared with horse blood. A streak of bacteria was added and incubated at 30 °C. Results were observed after 48 to 72 h and distinguished with a colorless area [32].

#### 2.7.6. Gelatinase

Nutrient agar medium containing 1% gelatin was inoculated with bacterial isolates and the plates were incubated at 30 or 37 °C for 2 to 5 days. A solution of mercury chloride was used to highlight the degradation of gelatin with a clear halo around the colonies [33].

#### 2.7.7. Chitinase

This test was carried out on nutrient agar supplemented with 1% chitin. After inoculation of bacteria in streaks, plates were incubated for 72 h at 30 °C. The appearance of light areas around the colonies would indicate the production of chitinase [34].

#### 2.7.8. Cellulase

The bacterial isolates were streaked on a plate and incubated for 72 h at 30 °C. Afterwards, 1% Congo red aqueous solution was added, and this allowed for the demonstration of cellulose decomposition after 15 min [35].

#### 2.7.9. Agarase

A qualitative test for the agarase enzyme was performed with iodine using Lugol’s staining process. Selected isolates were grown in ZMB (HiMedia, Kennett Square, PA, USA), pH 7.6 ± 0.2. An 8 mm well was cut in the agar, and 100 µL broth culture of each strain was added into the well and incubated at 30°C for 3 days. After incubation, the agarolytic activity was measured by adding 2 mL Lugol’s iodine on the entire surface of the plate and leaving it for 15 min [36].

Additionally, the agarolytic index (AI) was calculated as the ratio between the diameter of the clear zone and the colony. This index denotes the ability of the bacteria to produce agarase enzymes [36].

The calculating formula is shown below:

AI = Clear zone diameter (mm) − Colony diameter (mm)/Colony diameter (mm)

### 2.8. Antimicrobial Tests of CFF Associated Bacteria

To study the antibiotic activity, the spot method described by Fleming et al. [37] was used. A fresh culture of pathogenic bacteria (*Escherchia coli* (ATCC 14948), *Vibrio anguillarum* (ATCC 12964), *Vibrio alginolyticus* (ATCC 17749), *Pseudomonas aeruginosa* (ATCC 27853), *Salmonella typhymurium* (C52), and *Staphylococcus aureus* (ATCC 25923)), and the unicellular yeast (*Candida albicans* (ATCC 10231)) were prepared and cultured in 9 mL of marine broth and incubated at 30 °C for 24 h. After incubation, 9 mL of the target strain was diluted in Mueller Hinton Agar (MH) to 10^6^ CFU/mL. Then, the mixture was poured onto the MH layer. The Petri dishes were left at room temperature to allow for the drying of the strains. Then, fresh cultures of all isolated bacteria in nutrient broth were prepared. The spot test consisted of depositing a volume of 5 μL of the fresh culture from each strain on the dried plate. The same volume of sterile marine broth was also deposited as a negative control. The plates were then incubated at 30 °C for 24 h. The antibacterial activity was known from the appearance of light areas around the spots. The diameter of the inhibition zones was measured in millimeters, and the diameter of the spot was never considered in the expression of the results [38].

## 3. Results

### 3.1. Molecular Identification of Isolates

Culturable epiphytic bacteria from the CFF collected from the La Marsa site, were isolated. Fifty bacterial isolates were obtained from the surface of *Codium*. In addition, six bacterial isolates were isolated from the sediment and water. The obtained results show a higher number of culturable bacteria from the alga thallus compared to the sediment or the surrounding water.

**Figure 2:**
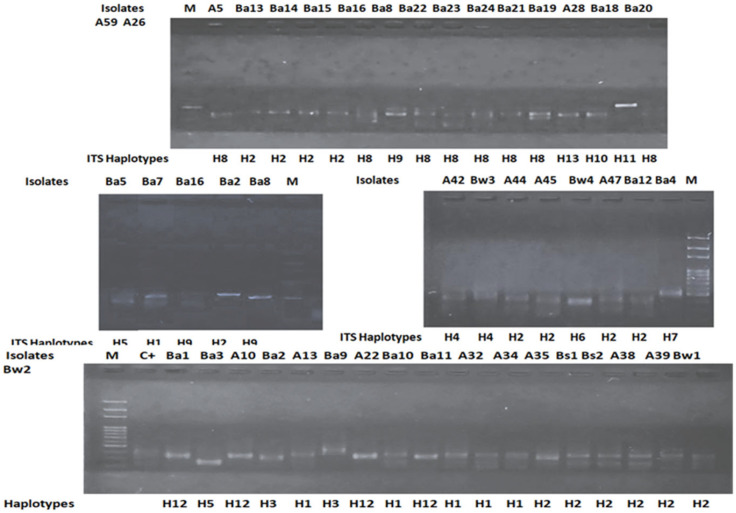
ITS-PCR fingerprinting patterns of epiphytic bacterial isolates resolved by agarose gel electrophoresis. Haplotypes:H1→H13, M: molecular size marker 100. H1 (A32,A34,A35,Ba7, Ba10), H2 (Bs1, Bs2,A38, A39,Bw1, Bw2, Ba13, Ba14,Ba15, Ba16, A47, A49, A44, A45), H3 (Ba6, Ba9), H4 (Bw3, A43), H5 (Ba3),H6 (Bw4), H7 (Ba4), H8 (A26,Ba2, Ba8, Ba19), H9 (Ba21, Ba22, Ba23, Ba24), H10 (Ba20), H11 (Ba17), H12 (Ba1,Ba5, A22, Ba11), and H13 (A28).

ITS-PCR fingerprinting was applied to assess the bacterial diversity of the selected isolates. The ITS profiles showed reproducible patterns consisting of 1 to 45 bands, with sizes ranging from 50 to about 1000 bp (Figure 2). Thirteen different ITS haplotypes were obtained. Six haplotypes, H5 (Ba3), H6 (Bw4), H7 (Ba4), H10 (Ba20), H11 (Ba17), and H13 (Ba18), were represented by one isolate. The following ITS profiles were found with at least two isolates: H3 (Ba6, Ba9), H4 (Bw3, A43), H8 (A26,Ba2, Ba8, Ba19), H9 (Ba21, Ba22, Ba23, Ba24), H12 (Ba1,Ba5, A22, Ba11) H1 (A32,A34,A35,Ba7,Ba10), H2 (Bs1, Bs2,A38, A39,Bw1, Bw2, Ba12, Ba13, Ba14,Ba15, Ba16, A47, A49, A44, A45).

**Figure 3:**
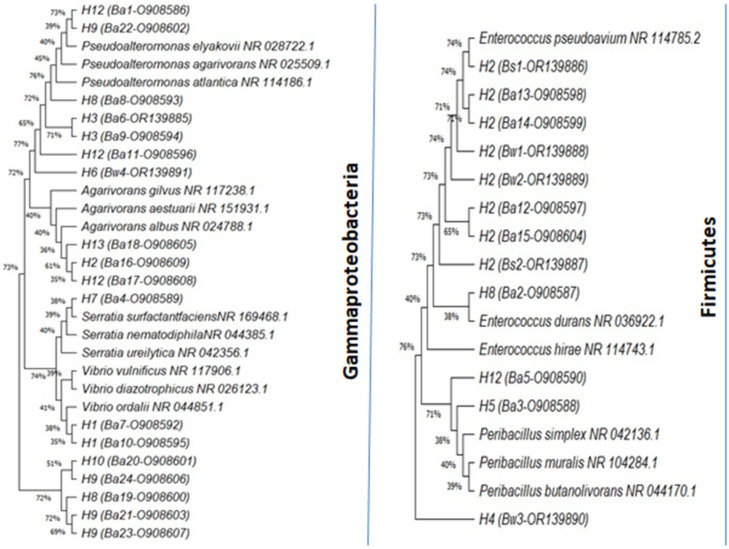
Neighbor-joining phylogenetic tree based on 16S rRNA gene sequence of bacteria isolated from the *Codium fragile* surface, surrounding water, and sediment.

A total of 30 bacterial isolates were selected and subjected to identification, characterization, and phylogenetic analysis, among which 24 were from the alga, 2 were from the sediment, and 4 were from the surrounding water (Table 2). Phylogenetic analysis revealed that the selected isolates belonged to two phylla, namely the Gammaproteobacteria (63%) and the Firmicutes (37%), showing 98–100% identity to the published species sequences. Based on 16S rRNA gene sequences compared to those of their close relatives, the differential alignment of bacterial isolates with different species was highlighted (Figure 3). Among the isolated strains, five strains assigned to genus Pseudoalteromonas included the following: *P. spiralis* (Ba1), *P. agarivorans* (Ba8), *P. piscicida* (Ba6 and Ba9), and *P. shioyasakiensis* (Ba11). The genus Pseudomonas included *P. khazarica* (Ba19, Ba20, Ba21, Ba23 and Ba24), while the genus Vibrio was represented by *V. anguillarum* (Ba7et Ba10) and *V. atlanticus* (Bw3). The genus Agarivorans included *A. litoreus* (Ba16), *A. abus* (Ba17), and *Agarivorans* sp. (Ba18), while the genus Peribacillus was represented by *P. frigoritolerans* (Ba3 and Ba5), and the genus Serratia was represented by *S. marcescens*. Finally, the genus Enterococcus was represented by *E. faecium* (Ba2, Ba12, Ba13, Ba14, Ba15, Bs1, Bs2, Bw1, and Bw2).

### 3.2. Antibiotic Resistant Profile of CFF Associated Bacteria

**Figure 4:**
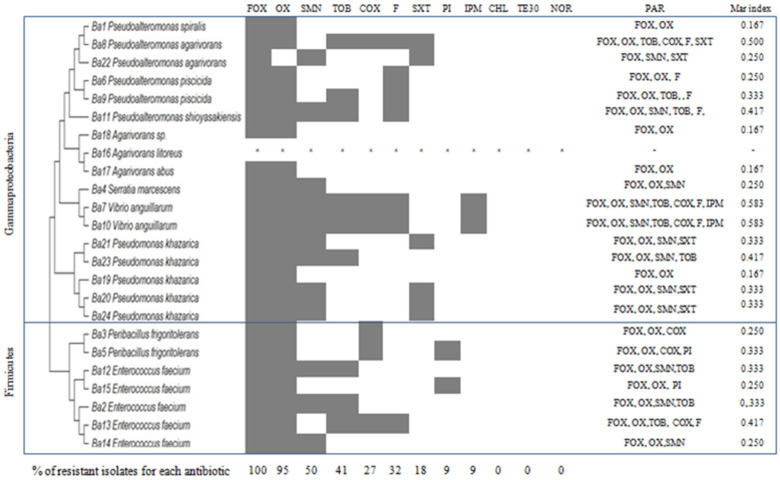
Antibiotic resistance profile of the CFF-associated bacteria: Ba1→Ba23; isolated from the alga surface, CHL: chloramphenicol, IPM: imipenem, SXT: trimethoprim/sulfamethoxazole, FOX: cefoxitin, COX: cefotaxime, TOB: tobramycin, SMN: streptomycin, F: nitrofurantoin, OX: oxacillin, TE30: tetracycline, PI: pipemidic acid, NOR: norfloxacin, PAR: Phenotype of antibiotic resistance.

The resistance profiles of 24 bacterial isolates from the CFF samples to 12 antibiotics are shown in Figure 4. A high percentage of the strains show antibiotic resistance to cefoxitin (90%) and oxacillin (87%), while a lower percentage of the strains show resistance to streptomycin (40%), tobramycin (37%), cefotaxime (30%), and to nitrofurantoin (27%). Most of the strains were resistant to imipenem (93% sensible), pipemidic acid (90%), and trimethoprim/sulfamethoxazole (87%), and all the isolated strains showed sensitivity to chloramphenicol, norfloxacin, and tetracycline. A phenotype involving various antibiotic resistances was observed. Ten dominant multi-resistance profiles were found for all bacterial isolates against six families of antibiotics (quinolones, penicillin, aminoglycoside, nitrofuran, pyridopyrimidine, and carbapenem). Over 40% of the strains showed a MAR index higher than 0.4. Isolates Ba7 and Ba10, closely related to *Vibrio anguillarum,* displayed the highest Mar index of 0.6 (Figure 4).

**Figure 5:**
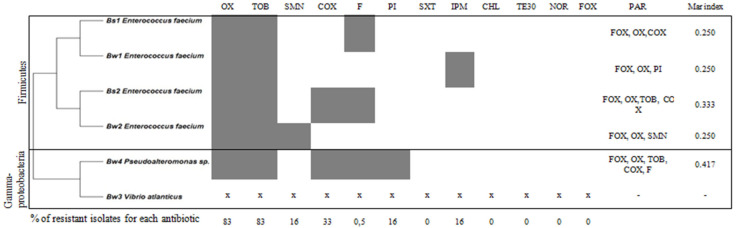
Antibiotic resistance profile of associated bacteria Bs1→Bs2: isolated from sediment, Bw1→w4; isolated from water. CHL: chloramphenicol, IPM: imipenem, SXT: trimethoprim/sulfamethoxazole, FOX: cefoxitin, COX: cefotaxime, TOB: tobramycin, SMN: streptomycin, F: nitrofurantoin, OX: oxacillin, TE30: tetracycline, PI: pipemidic acid, NOR: norfloxacin, PAR: Phenotype of antibiotic resistance.

The antibiotic resistance profiles of the associated bacteria isolated from sediment and from water are shown in Figure 5. All isolates revealed sensitivity to cefoxitin, norfloxacin, tetracycline, chloramphenicol, and trimethoprim/sulfamethoxazole, with *Pseudoalteromonas* sp. presenting the highest MAR index with 0.4.

### 3.3. Screening of Qualitative Enzyme Production of CFF Associated Bacteria

**Figure 6:**
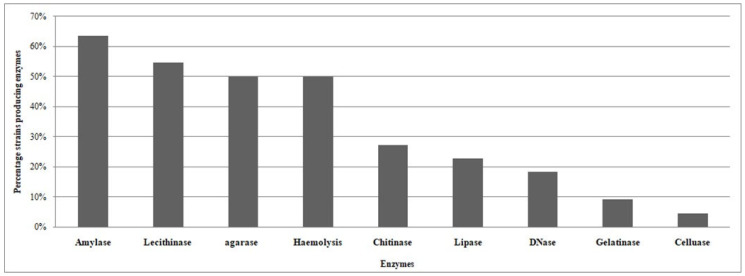
Percentage of the CFF isolated strains producing enzymes.

Table 3 presents an enzymatic production profile of the CFF-associated isolates. Most of the isolates show the ability to produce enzymes, with 71% producing more than one enzyme. Three strains, Ba8 and Ba22 (closely related to *Pseudoalteromonas agarivorans*), and Ba23 (closely related to *Pseudomonas khazarica*), showed the ability to produce at least five enzymes, while three CFF-associated strains, Ba2 (*Enterococcus faecium*), Ba3 (*Peribacillusfrigoritolerans*), and Ba10 (*Vibrio anguillarum*), had the ability to produce 50% of the tested enzymes. The most produced enzymes by the CFF-associated strains were amylase, lecithinase, and agarase with percentages of 64%, 55%, 50%, respectively (Figure 6). The most productive genera among isolates were the *Pseudoalteromonas*, *Pseudomonas*, *Enterococcus* and *Vibrio* strains. These strains had the ability to produce chitinase, amylase, and cause hemolysis, while *Agarivorans* isolates had the ability to produce agarase and lecithinase.

### 3.4. Qualitative Assays of Agarase Enzyme Production

**Figure 7:**
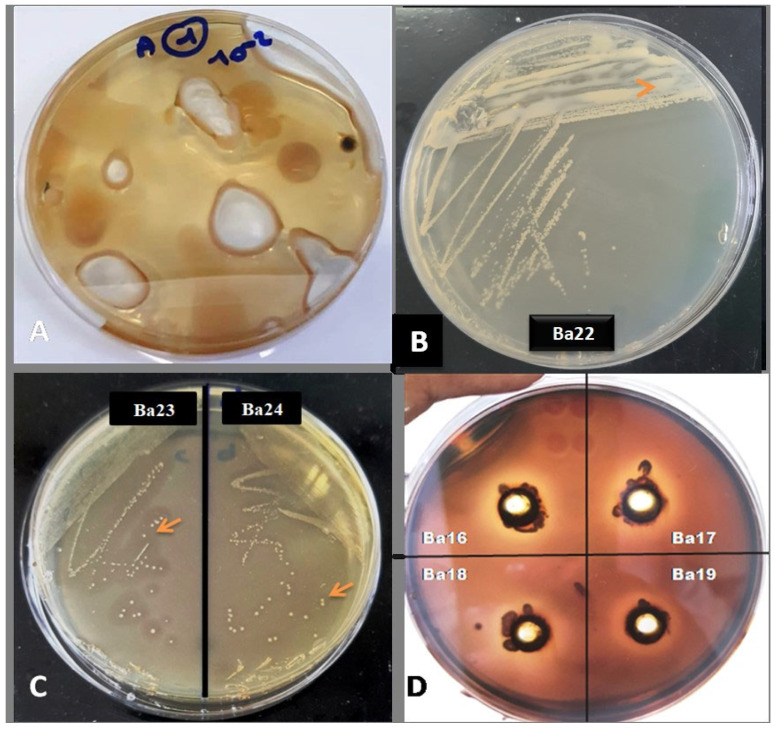
Agar liquefying by agarolytic bacterium strain after 5 days of incubation in solid medium (**A**), Clearance zone around the colony of the strain (Ba23) and (Ba24) (**B**), agarolytic bacterial colonies (Ba 22) showing liquefaction (**C**), and halo zone for the qualitative test for agarase enzymes performed with iodine using Lugol’s staining process (**D**); arrows pointing to clearance zones.

Nine strains demonstrate important agarose activity as shown in Figure 7. All the isolates (Ba16, Ba17, Ba18, Ba19, Ba20, Ba21, Ba22, Ba23, and Ba24) showed a clear light-colored zone around the culture when the dark brown iodine was poured on the culture plates. The potential of the agarolitic bacteria in producing agarase enzymes can be seen qualitatively by calculating their agarolitic index. Table 4 shows the agarolitic index enzyme activity obtained for the CFF bacterial isolates. A clear zone forming around the colony with a different halo upon the addition of drops of iodine solution would demonstrate the enzymatic activity of extracellular agarose produced by agarolitic isolates from the CFF (Table 3). Ba16, Ba17, and Ba18, closely related to *Agarivorans litoreus*, *Agarivoransabus*, *Agarivorans* sp., respectively, gave the higher agarolitic index.

### 3.5. Antimicrobial Activity of CFF Associated Bacteria

Antimicrobial activity observed for the isolated strains is shown in Table 5. Out of the 24 strains, 9 displayed antimicrobial activity against *E. coli*, consisting of 8 Gammaproteobacteria (Ba1, Ba3, Ba4, Ba7, Ba8, Ba10, Ba11 and Ba12) and 9 Firmicutes (Ba12).Conforming to the results, the strongest antimicrobial activity was obtained for the strains Ba1 (closely related to *Pseudoalteromonas spiralis*), Ba12 (closely related to *Enterococcus faecium*), and Bw4 (closely related to *Pseudoalteromonas* sp.) with an inhibition against *E. coli* showing a diameter of more than 20 mm. The isolate Ba4 (closely related to *Serratia marcescens*) had the largest antimicrobial activity, inhibiting the six pathogenic bacteria tested as well asthe yeast. Only one strain, Ba4 (closely related to *Serratia marcescens*), displayed a wide screen of antimicrobial activity, showing positive inhibition of all the tested pathogenic species.

## 4. Discussion

Our findings suggest that *Codium fragile* ssp. *fragile* hosts a varied community of cultivable bacteria, affirming that the CFF thallus is indeed a conducive matrix for bacterial accumulation and growth. Phylogenetic analysis revealed that the selected isolates represented 98–100% identity to the published species sequences. However, six isolates’ sequences had a percentage identity below 98.7% (Ba16, Ba17, Ba18, Ba20, Ba24 and Bw4), which suggests that these may be new species [39]. The majority of the isolated strains belong to the phyla Gammaproteobacteria. These findings are in accordance with several studies showing that Proteobacteria phyla are common members of macroalgal bacterial communities [40], with dominance in the surface of green algae [41]. A study on *Codium tomentosum* showed that the majority of the bacterioflora is largely represented by Proteobacteria (94.6%) and secondarily by Bacteroidetes, Spirochaetes, and Firmicutes [42].

At the genus level, it is observed that *Pseudoalteromonas*, *Pseudomonas*, and *Enterococcus* were the most abundant genera in the current study, where bacteria were collected from the surface of the entire thallus. Epiphytic bacteria like *Azotobacter* have been evidenced at the surface of the utricles of *Codium fragile* [43], while cyanobacterial colonies may develop between the bases of the utricles in *Codium decorticatum* [44]. Illustrations with scanning electron microscopy revealed the presence of bacteria, mainly of the *Bacillus* type, at the level of the apical region of the utricle [45].

In the present study, a phenotype involving various antibiotic resistances was observed. Ten dominant multi-resistance profiles were found for all the bacterial isolates against six families of antibiotics (quinolones, penicillin, aminoglycoside, nitrofuran, pyridopyrimidine, and carbapenem). The MAR index of the majority of the bacteria ≥ 0.2, as mentioned in previous studies, increases the consumption of antibiotics and thus contributes to an increase in environmental waste [46]. The ecological basis of antibiotic resistance is the adaptation of bacteria to a polluted environment. Bacteria present in these contaminated aquatic matrices share or exchange transferable elements of DNA with other bacteria, and this can occur between different bacterial species [47]. Seawater is often contaminated with drug residues [48]. This contamination encourages the spread of resistance and even of multi-resistant bacteria [49]. Few data are available on multidrug resistance patterns of marine vegetation-associated bacteria. Resistance to Aztreonam, Ceftazidime, Amoxicillin, and Rifampicin has been reported for epi-endophytic bacterial strains isolated from *Posidonia oceanica* seagrass from the eastern coast of Tunisia, with a high MAR index of 0.67 [50].

The isolates investigated in this study showed diversified enzymatic activity. The majority of the isolates associated with CFF have the capacity to produce amylase. Amylases have the potential to be used in a wide range of industrial processes; for example, a large variety of microbial α-amylases have applications in food, textile, paper, and detergent industries [51]. CFF-associated bacterial strains have the ability to produce Lecithinases (59%). Lecithinases are also capable of hemolysis, and they work synergistically with phospholipases to hydrolyze lipids and lecithin. In this study, the average of chitinase-positive strains appears to be 36.4%, particularly considering those isolates obtained from CFF, the surrounding water, and sediment. Degradation of chitin in the aquatic biosphere is a very active and efficient process mainly carried out by bacteria. It is well known that chitinolytic bacteria are ubiquitous in marine environments. However, in our study, we found chitinolytic strains in sediment and in water. Chitinolytic enzymes have a wide range of potential applications in biotechnology, and the search for innovative chitinolytic bacteria continues to be a fascinating subject. Some applications of chitinases in the food and wine industries have been successfully tested at the laboratory level [52]. Only a few strains were found positive for cellulase. However, due to the crucial applications of these enzymes, positive strains could have highly valuable biotechnological uses [53].

Currently, a number of microorganisms have been reported to secrete agarase, and are mainly in a marine environment, either in the sea water, marine sediments, or associated to red algae [54,55]. An agar-degrading bacteria, *Agarivorans* sp., has been isolated from the red alga *Grateloupia filicina*. The enzyme has been characterized as part of the glycoside hydrolase family, β-agarase. By degrading agar, the enzyme produced neoagaro-oligosaccharides with biological properties [56]. Agarase is primarily used to produce oligosaccharides from agar. Agar-derived oligosaccharides have many functions, including hepatoprotective potential [57], anti-oxidation [58] and have potential applications in the food, cosmetic [59], and medical industries. In addition, agarase can also be used to degrade the cell walls of seaweed to generate protoplasts [60,61]. Agarase has also been reported to be used to recover DNA from agarose gel [62,63]. Recently, efforts have been made to find even more active agarases in the environment.

All the isolates grown on solid media were aerobic, showed clear bright zones around the colonies which degraded agar as shown by the iodine test, and showed pits on the surface of the agar medium. It is concluded that the higher agarolitic activity detected from isolates Ba16, Ba17, and Ba18 had an agarolitic index equivalent to 2.38, 2.13, and 2.38, respectively. Our results are comparable to those found by [27], who obtained an agarolytic index of 3.75 mm and 2.53 mm for isolates Sg8 and A13 respectively, isolated from marine algae in India. Agarolitic bacteria could be divided into two groups, namely those which only softened the agar and those which caused extensive liquefaction of agar. The isolated strains of agarolytic bacteria produced at least two enzyme complexes, one cell-free and the other cell-bound, which hydrolyzed agar with the formation of oligosaccharides [64]. A new agarase was thus purified from an agarolytic bacterium, *Bacillus megaterium,* a novel agar-degrading bacterium isolated from marine sediment [65]. Agar-degrading organisms have been isolated from a wide range of environments, including seawater, marine sediments, marine algae, marine mollusks, fresh water, and soils. Agarase activity has been investigated in various bacteria, including the genus *Alteromonas*, *Pseudomonas*, *Vibrio*, *Cytophaga*, *Agarivorans*, *Thalassomonas*, *Pseudoalteromonas*, *Bacillus,* and *Acinetobacter* [66].

In the present work, the agarolitic isolates were susceptible to Chloramphenicol, imipenem, Trimethoprim/sulfamethoxazole, Cefotaxime, Tobramycin, Streptomycin, Nitrofurantoin, Tetracycline, Pipemidic acid, and Norfloxacin. All the strains isolated from CFF were sensitive to Oxacillin and Cefoxitin. In particular, theisolated agarolytic strains were susceptible to penicillin, kanamycin, nitrofurantoin, and tobramycin [67]. Representatives of the aerobic genus *Agarivorans*, which is related to Proteobacteria, may generate agarase and catalyze the hydrolysis of agar. A unique strain WH0801T was recently isolated from the surface of seaweed in the shallow coastal region of Weihai, China, for its agarolytic activity and has been suggested as a new species, *Agarivorans gilvus* [68]. Agarolytic bacteria are likely involved in the degradation of algal polysaccharides. The presence of these bacteria may play an important role in the nutrient cycling of marine ecosystems and can have biotechnological applications in the production of agar-based products. Agarases are hydrolytic enzymes used in biotechnological and commercial applications including the following: decomposing algal polysaccharides, the creation of simple sugars, biofilm removal in bioreactors, making bread and low-calorie foods in the food sector [69], as well as applications in cosmetics, and medical industries [60]. Agarase can be used for molecular biology applications such as the extraction of DNA or RNA fragments from agarose gel [70]. Some agar oligosaccharides obtained with agarase enzyme have anti-oxidative, antibacterial, antimutagenic, and immune-modulating properties [71].

In this study, 37% of the isolates displayed antibacterial activity, which is in accordance with findings from research conducted along the coast of Scotland, where 22% of the isolated strains from *Codium fragile* presented antimicrobial activity [72].The strain Ba4 (closely related to *Serratia marcescens*) was the most active strain, inhibiting the growth of all the pathogenic species used. Interestingly, a previously isolated strain from the genus *Serratia*, associated with the coralline red alga *Amphiroa anceps*, demonstrated antimicrobial activity [4]. The isolate showed antibacterial activity against *E. coli*, *Staphylococcus aureus,* and *Klebsiella* sp., and exhibited antifungal properties. Besides *Serratia nematodiphila* and *Serratia marcescens* were isolated from the Malaysian marine environment and have been reported to produce strong antimicrobial compounds acting against *Staphylococcus aureus* and *Candida albicans.* Nevertheless, the isolate did not show any activity against *E. coli* [73,74]. Arivuselvam et al. [74] reported that the *Serratia marcescens* JSSCPM1 strain exhibited significant antibacterial activity against the Gram-negative bacterial strains *E. coli* NCIM 2065, *K. pneumoniae* NCIM 2706, and *P. aeruginosa*.

Species of the genus *Pseudoalteromonas* are often associated with eukaryotic hosts [75,76]. The presence of the *Pseudoalteromonas* species in diverse habitats worldwide suggests that their adaptive and survival strategies are varied, efficient, and hold significant potential for both fundamental and practical research. Many *Pseudoalteromonas* species have been demonstrated to produce antibacterial products which appear to help them in the colonization of surfaces, including those of their hosts [76]. *Pseudoalteromonas* species display a broad range of antibiotic, agarolytic, algicidal, and antifouling effects. The production of agarases, toxins, bacteriolytic substances, and other enzymes by many *Pseudoalteromonas* species may assist the bacterial cells in their competition for nutrients and space, as well as in their protection against predators grazing at surfaces [77]. Furthermore, the marine bacterium, *Pseudoalteromonas phenolica* sp. nov. O-BC30T was active against themethicillin-resistant *Staphylococcus aureus* and the hypersensitive *Escherichia coli* mutant (KO 1489) [78].

In this study, the isolate Ba12, identified as closely related to *Enterococcus faecium*, demonstrated antibacterial activity against *E. coli. Enterococcus* is also known to be one of the most commonly used lactic acid-producing bacteria and has since become a focus for its use in commercially farmed aquatic species [79]. Moreover, probiotics are used to improve water quality and control of bacterial infections, can improve the digestibility of nutrients, increase tolerance to stress, and encourage reproduction [80]. Thus, our findings can be of great interest for aquaculture industry to develop sustainable commercial probiotic products prepared from the *Enterococcus* species isolated from algae.

Concerning the antimicrobial activity of the associated bacteria in same area, the results of the present study is in agreement with that found by [12] and [81], which showed that several bacterial strains isolated from the *Ulva* surface have an important antimicrobial activity.

## 5. Conclusions

*Codium fragile* is a marine alga widely distributed in the Mediterranean Sea, particularly along the Tunisian coast. This alga has been found to harbor diverse bacterial communities, including agarolytic bacteria, a polysaccharide commonly found in the cell walls of red macroalgae which are known to degrade agar. The associated bacterial communities of *Codium fragile* ssp. *fragile* collected from the northern Tunisian coast are diverse and displayed different enzymatic profiles. The use of culture-dependent and culture-independent methods provides a more comprehensive understanding of the bacterial communities associated with marine algae and their potential biotechnological applications. Culturable associated bacteria presented a multidrug resistance profile, suggesting their adaptation and ability to encode antibiotic resistance genes. Further studies are needed to explore the ecological and biotechnological significance of these bacterial communities.

## Figures and Tables

**Table 1:** The antibiotics and concentrations used in this study.

Class	Abbreviation	Antibiotic	Amount (µg)
Phenicol	CHL	Chloramphenicol	30
Carbapenem	IPM	Imipenem	10
Diaminopyrimidines	SXT	Trimethoprim/Sulfamethoxazole	1.25/23.75
Quinolones	FOX	Cefoxitin	30
Quinolones	COX	Cefotaxime	5
Aminoglycoside	TOB	Tobramycin	10
Aminoglycoside	SMN	Streptomycin	10
*Nitrofuran*	F	Nitrofurantoin	300
Penicillin	OX	Oxacillin	1
	TE30	Tetracycline	30
Pyridopyrimidine	PI	Pipemidicacid	20
Quinolone	NOR	Norfloxacin	5

**Table 2:** Epibiotic bacteria isolated from the invasive macroalga *Codium fragile* ssp. *fragile* surface, sediment, and surrounding water.

ITS Haplotype	Isolates	Ox	Cat	Closestspecies in NCBI	Size (bp)	Identity (%)	Accession Number	Phylum
H1	Ba10	+	+	*Vibrio anguillarum*	1050	99.62	ON908595	γp
H1	Ba7	+	+	*Vibrio anguillarum*	1111	99.82	ON908592	γp
H2	Ba12	+	+	*Enterococcus faecium*	1140	99.56	ON908597	F
H2	Ba13	+	+	*Enterococcus faecium*	1136	99.74	ON908598	F
H2	Ba14	+	+	*Enterococcus faecium*	1122	99.91	ON908599	F
H2	Ba15	+	+	*Enterococcus faecium*	1005	99.80	ON908604	F
H2	Ba16	+	+	*Agarivorans* *litoreus*	1056	94.33	ON908609	γp
H2	Bs1	+	+	*Enterococcus faecium*	1157	98.88	OR139886	F
H2	Bs2	+	+	*Enterococcus faecium*	1128	99.65	OR139887	F
H2	Bw1	+	+	*Enterococcus faecium*	1110	99.91	OR139888	F
H2	Bw2	+	+	*Enterococcus faecium*	1145	99.74	OR139889	F
H3	Ba6	+	+	*Pseudoalteromonas piscicida*	1124	99.82	OR139885	γp
H3	Ba9	+	+	*Pseudoalteromonas* *piscicida*	1086	99.72	ON908594	γp
H4	Bw3	+	+	*Vibrio atlanticus*	1104	99.37	OR139890	γp
H5	Ba3	+	+	*Peribacillus* *frigoritolerans*	1140	99.22	ON908588	F
H6	Bw4	+	+	*Pseudoalteromonas* sp.	988	90.26	OR139891	γp
H7	Ba4	+	+	*Serratia marcescens*	1129	99.56	ON908589	γp
H8	Ba8	+	+	*Pseudoalteromonas* *agarivorans*	1079	100	ON908593	γp
H8	Ba19	+	+	*Pseudomonas khazarica*	1139	98.43	ON908600	γp
H8	Ba2	+	+	*Enterococcus faecium*	1101	98.73	ON908587	F
H9	Ba21	+	+	*Pseudomonas khazarica*	1104	98.46	ON908603	γp
H9	Ba22	+	−	*Pseudoalteromonas* *agarivorans*	1129	99.47	ON908602	γp
H9	Ba23	+	+	*Pseudomonas khazarica*	1072	98.42	ON908607	γp
H9	Ba24	+	+	*Pseudomonas khazarica*	1109	97.93	ON908606	γp
H10	Ba20	+	−	*Pseudomonas khazarica*	784	97.96	ON908601	γp
H11	Ba17	+	+	*Agarivorans* *abus*	1056	94.33	ON908608	γp
H12	Ba1	+	+	*Pseudoalteromonas spiralis*	1333	99.38	ON908586	γp
H12	Ba5	+	+	*Peribacillus* *frigoritolerans*	1117	99.91	ON908590	F
H12	Ba11	−	+	*Pseudoalteromonas* *shioyasakiensis*	1011	98.81	ON908596	γp
H13	Ba18	+	+	*Agarivorans* sp.	1009	92.54	ON908605	γp

Isolates: (Ba1→Ba23: isolated from alga surface, Bs1→Bs2: isolated from sediment, Bw1→Bw4: isolated from water), OX: oxidase, Cat: catalase, bp: base pair, γp: Gammaproteobacteria, F: Firmicutes. (+) presence of activity, (−) absence of activity.

**Table 3:** Screening of qualitative enzymatic production of CFF-associated bacteria, sediment, and surrounding water.

REF	Isolates	Amylase	Lecithinase	Hemolysis	Chitinase	DNase	Lipase	Gelatinase	Cellulase	Agarase
CFF-associated bacteria										
Ba1	*Pseudoalteromonas* *spiralis*	−	−	+	−	−	+	−	−	−
Ba2	*Enterococcus aecium*	+	+	+	−	−	+	−	−	−
Ba3	*Peribacillus* *frigoritolerans*	+	+	−	−	+	+	−	−	−
Ba4	*Serratia marcescens*	+	−	+	−	−	−	−	−	−
Ba5	*Peribacillus* *frigoritolerans*	+	−	+	−	−	−	−	−	−
Ba8	*Pseudoalteromonas* *agarivorans*	+	+	+	+	−	−	+	−	−
Ba9	*Pseudoalteromona spiscicida*	+	−	+	−	+	−	−	−	−
Ba10	*Vibrio anguillarum*	+	+	−	+	−	−	+	−	−
Ba11	*Pseudoalteromonas* *shioyasakiensis*	+	−	−	−	−	−	−	−	−
Ba12	*Enterococcus faecium*	x	x	x	x	x	x	x	x	−
Ba13	*Enterococcus faecium*	+	+	+	−	−	−	−	−	+
Ba14	*Enterococcus faecium*	+	−	+	+	−	−	−	−	+
Ba15	*Enterococcus faecium*	−	−	−	−	−	−	−	−	+
Ba16	*Agarivorans* *litoreus*	−	−	−	−	−	−	−	−	+
Ba17	*Agarivorans* *abus*	−	−	−	−	−	−	−	−	+
Ba18	*Agarivorans* sp.	−	+	−	−	−	−	−	−	+
Ba19	*Pseudomonas khazarica*	−	−	−	−	−	−	−	−	+
Ba20	*Pseudomonas khazarica*	−	+	+	−	−	−	−	−	+
Ba21	*Pseudomonas khazarica*	−	+	−	+	−	−	−	−	+
Ba22	*Pseudoalteromonas* *agarivorans*	+	−	+	+	−	+	−	+	+
Ba23	*Pseudomonas khazarica*	+	+	+	+	+	−	−	−	+
Ba24	*Pseudomonas khazarica*	+	+	−	−	−	−	−	−	+
Sediment and surrounding water										
Bs1	*Enterococcus faecium*	−	−	−	−	−	−	−	−	−
Bs2	*Enterococcus faecium*	+	−	−	+	−	−	−	−	−
Bw1	*Enterococcus* *faecium*	−	−	−	−	+	−	−	−	−
Bw2	*Enterococcus faecium*	+	+	−	+	+	−	−	−	−
Bw3	*Vibrio atlanticus*	+	−	−	−	−	−	−	−	−
Bw4	*Pseudoalteromonas* sp.	+	−	+	−	+	−	−	−	−

Ba1→Ba24; isolated from alga surface, Bs1→Bs2: isolated from sediment, Bw1→Bw4; isolated from water, +: positive response, −: negative response, x: not tested.

**Table 4:** Agarolitic index halo zone of a qualitative test for the agarase enzyme.

Isolates	Agarolitic Index
Ba16	2.38 ± 0
Ba17	2.13 ± 0
Ba18	2.38 ± 0
Ba19	1.13 ± 0
Ba20	0.63 ± 0
Ba21	1.13 ± 0
Ba22	1.13 ± 0
Ba23	0.71 ± 0.57
Ba24	0.88 ± 0

**Table 5:** Antimicrobial activity of bacterial isolates from CFF surface, surrounding water, and sediment.

Isolates	Ref	*Pathogenic bacteria*						Yeast
		E.c	V.an	V.al	P.a	S.t	S.a	C.a
*Pseudoalteromonas spiralis*	Ba1	23 mm ±1.2	0	0	0	0	0	0
*Peribacillus frigoritolerans*	Ba3	10 mm ±1.5	0	0	0	0	0	0
*Serratia marcescens*	Ba4	8 mm ±0.6	10 mm ±1	8 mm ±0.6	8 mm ±0.6	10 mm ±1	12 mm ±0.6	13 mm ±1
*Vibrio anguillarum*	Ba7	13 mm ±0.6	0	0	0	0	0	0
*Pseudoalteromonas agarivorans*	Ba8	15 mm ±2.1	0	0	0	0	0	0
*Vibrio anguillarum*	Ba10	13 mm ±1.5	0	0	0	0	0	0
*Pseudoalteromonas shioyasakiensis*	Ba11	13 mm ±0.6	0	0	0	0	0	0
*Enterococcus faecium*	Ba12	22 mm ±2.6	0	0	0	0	0	0
*Pseudoalteromonas* sp.	Bw4	22 mm ±2.1	0	0	0	0	0	0

Ba: bacteriaisolatedfromalga surface, Bw: isolatedfrom water, E.c: Escherichia coli, V.an: Vibrio anguillarum, V.al: Vibrio alginolyticus, P.a: Pseudomonas aeruginosa, S.t: Salmonella typhimurium, S.a: Staphylococcus aureus, C.a: Candida albicans.

## Data Availability

The raw data supporting the conclusions of this article will be made available by the authors upon request.
